# Efficient overexpression and purification of severe acute respiratory syndrome coronavirus 2 nucleocapsid proteins in *Escherichia coli*

**DOI:** 10.1042/BCJ20240019

**Published:** 2024-05-24

**Authors:** Emma L. Brudenell, Manoj B. Pohare, Domen Zafred, Janine Phipps, Hailey R. Hornsby, John F. Darby, Junxiao Dai, Ellen Liggett, Kathleen M. Cain, Perdita E. Barran, Thushan I. de Silva, Jon R. Sayers

**Affiliations:** 1 Sheffield Institute for Nucleic Acids and Florey Institute, Section of Infection and Immunity, Division of Clinical Medicine, School of Medicine and Population Health, The University of Sheffield, Beech Hill Road, Sheffield S10 2RX, U.K.; 2 Michael Barber Centre for Collaborative Mass Spectrometry, Department of Chemistry, Manchester Institute of Biotechnology, The University of Manchester, 131 Princess Street, Manchester M1 7DN, UK

**Keywords:** ELISA, expression, nucleocapsid, recombinant protein, ribonucleoproteins, SARS-CoV-2

## Abstract

The fundamental biology of severe acute respiratory syndrome coronavirus 2 (SARS-CoV-2) nucleocapsid protein (Ncap), its use in diagnostic assays and its potential application as a vaccine component have received considerable attention since the outbreak of the Covid19 pandemic in late 2019. Here we report the scalable expression and purification of soluble, immunologically active, SARS-CoV-2 Ncap in *Escherichia coli*. Codon-optimised synthetic genes encoding the original Ncap sequence and four common variants with an N-terminal 6His affinity tag (sequence MHHHHHHG) were cloned into an inducible expression vector carrying a regulated bacteriophage T5 synthetic promoter controlled by *lac* operator binding sites. The constructs were used to express Ncap proteins and protocols developed which allow efficient production of purified Ncap with yields of over 200 mg per litre of culture media. These proteins were deployed in ELISA assays to allow comparison of their responses to human sera. Our results suggest that there was no detectable difference between the 6His-tagged and untagged original Ncap proteins but there may be a slight loss of sensitivity of sera to other Ncap isolates.

## Introduction

**Figure 1.**
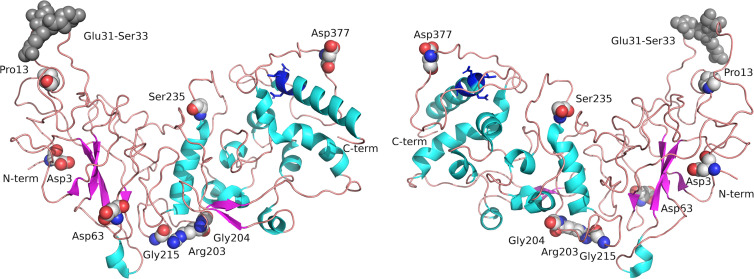
Structure of nucleocapsid protein. The structure of Ncap is shown rendered from 8FD5 in two views rotated 180° around a central *y* axis. Helical regions are shown in cyan and β strands in magenta connected by loops possessing poorly-defined secondary structure. N and C terminal regions are labelled. Mutated residues in the proteins studied here are labelled and shown as spheres (carbon grey, oxygen red, nitrogen blue). The CASP6 cleavage site is shown in dark blue sticks (residues 399–402). Compared with the wild-type sequence, B.1.1 contains two amino acid variations, (Arg203Lys, and Gly204Arg), Alpha contains four (Asp3Leu, Arg203Lys, Gly204Arg and Ser235Phe), as does Delta (Asp63Gly, Arg203Met, Gly215Cys and Asp377Tyr) while Omicron has a deletion of Glu31-Ser33 (dark grey spheres) and two amino acid substitutions (Arg203Lys and Gly204Arg).

The archetypal severe acute respiratory syndrome coronavirus 2 (SARS-CoV-2) virus N gene encodes a nucleoprotein, also known as a nucleocapsid (Ncap) or N protein (N), consisting of 419 amino acids comprised of two domains (Uniprot entry P0DTC9) [1]. The N-terminal contains a predominance of β-strand and coil structure with little helical content. In contrast with this, the C-terminal domain contains eight helical regions and a very short antiparallel two-stranded β-sheet feature (Figure 1) [2]. These domains are linked by a ∼70 amino acid unstructured linker region which appears to interact with non-structural protein 3 (NSP3) [3], a polypeptide consisting of 1945 amino acids which is involved in formation of viral replication-transcription complexes [4] as well as the membrane anchored viral M protein [5].

Both the N-terminal RNA-binding and C-terminal dimerisation domains appear to interact with viral ribonucleic acid [5]. The protein is subject to post-translational modifications such as phosphorylation [6] and host-mediated proteolysis by the cysteine-aspartic protease caspase 6 [7]. This releases fragments that reduce the host's inflammatory response by antagonising interferon gamma production [8].

Recombinant Ncap protein has also been used to facilitate development of both ELISA [9–12] and lateral flow diagnostics systems [12–15]. The SARS-CoV-2 Ncap protein has been suggested as a possible vaccine candidate [16,17] and it has been demonstrated that immunisation with recombinant Ncap produced in *Escherichia coli* induced an antibody response in the lungs of rats [18]. Furthermore, next-generation vaccines are being developed that will express both Spike and Ncap proteins using adenovirus vectors [19] or using encapsulated mRNA approaches [20].

Since the first SARS-CoV-2 sequence was reported [1,3], much attention has focused on mutations in the Spike protein and their impact on vaccine efficacy has been reviewed extensively [21–23]. However, mutations in the N gene encoding Ncap are also of interest as these may impact on pathogenicity [4,24] and on diagnostic test efficacy. For example, the B.1.1 lineage [25,26] arose in early 2020 which contained a double mutation in Ncap, Arg203Lys and Gly204Arg, [27] which spread throughout Europe and beyond. Further examples include Alpha (B.1.1.7) [28], Delta (B.1.617) [29] and Omicron (B.1.1.529) [30] amongst others [26,31,32]. Considerable ongoing sequence surveillance seems certain to identify further new variants [33–37]. Two studies report on whether Ncap protein sequence variation leads to reduced sensitivity for some rapid antigen tests (RATs). Hagag et al. [38] found that the Arg203Met mutation, present in Delta, led to complete loss of detection in the 4 RATs tests they examined, while a study examining 11 different commercially available tests found no cause to suspect that common variants circulating at the time would be less effectively detected [39].

Here we report the expression, purification and characterisation of recombinant Ncap proteins using codon-optimized synthetic genes in *E. coli*. Native (untagged) and Ncap adorned with an N-terminal tag consisting of six histidine residues were all produced in soluble form, purified and their responses to human sera compared. Tagged Ncap variants including B.1.1, Alpha, Delta and Omicron were also produced and examined.

## Results and discussion

### Potential impact of mutation on Ncap function

**Figure 2.**
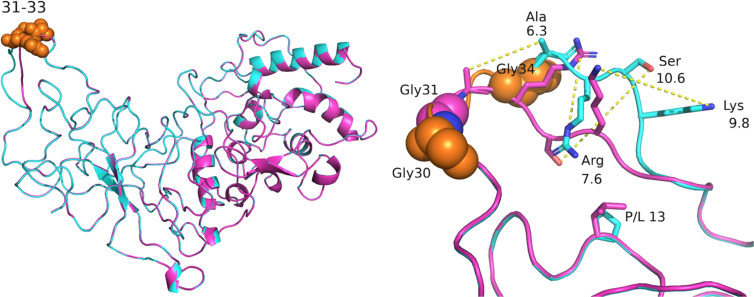
Impact of Omicron variant mutations on Ncap structure. Wild type Ncap structure (cyan cartoon, based on PDB entry 8FD5) was aligned with the predicted structure of the Omicron variant (magenta) as shown in the left panel. The two structures overlay well except in the region of the Omicron deletion. In Omicron, the deletion of residues 31–33 results in two adjacent glycine residues (residue 30, orange spheres and 31, previously 34 in the WT, magenta spheres). The side chains of residues Ala-Arg-Ser-Lys (35–38 in the WT sequence) show the largest structural changes (dotted yellow line show displacement in Å). Mutation of proline 13 to leucine (P/L 13) is predicted to have minimal structural impact.

We modelled the impact of mutations on local protein structure (Figure 2 and Supplementary Figure S1). Unsurprisingly, deletion of residues 31–33 in the Omicron variant results in the largest apparent structural changes (Figure 2) but the Pro13-Leu substitution appeared to cause little local structural perturbation to the modelled, energy minimised structures. Other substitutions also seem likely to have an obvious impact. For example, the Asp3Leu and Ser235Phe substitutions in Alpha Ncap result in loss of two charge-charge (ion pair) interactions with arginine residues 88 and 92 in the former as well as loss of a hydrogen bond between the serine hydroxyl group and backbone carbonyl of amino acid 189 in the latter (Supplementary Figure S1A). Similarly, the Arg203Lys/Gly204Arg double substitution present in B.1.1, Alpha and Omicron, potentially result in additional hydrogen bonds formed between the side chains of Arg204 (with amide oxygen of residue Ser202) and Lys203 (the backbone of Asp215) as shown (Supplementary Figure S1B). In contrast, amino acid substitutions Asp63Gly and Arg 203Met in Delta Ncap both result in loss of a charged partner in ion pair interactions (with Lys65 and Asp216, respectively) compared with WT Ncap as (Supplementary Figure S1C).

In addition to the full-length CryoEM structure [2], several X-ray structures have been reported for isolated N or C terminal domains [40–44], and one NMR structure of the ‘linker region’, residues 191–262 [3] which shows interactions with the viral protein NSP3.

**Figure 3.**
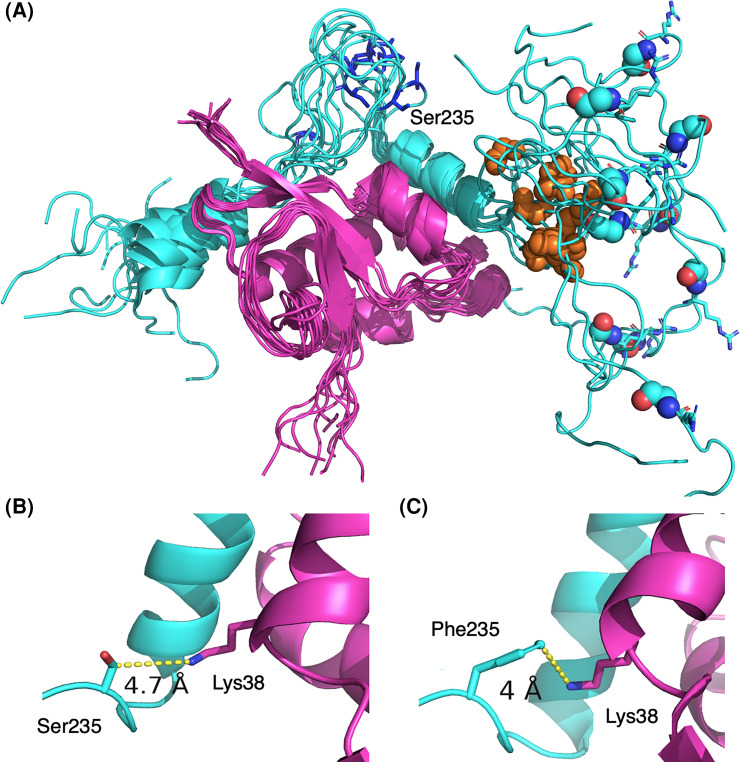
Interactions between the Ncap linker region and NSP3. (**A**) The ensemble NMR structure of Ncap (cyan cartoon) in complex with NSP3 (PDB code 7PKU). Glycines 204 and 215 are shown as spheres, in cyan and orange respectively. Ser235 is labelled and shown as blue sticks and Arg203 shown as cyan sticks. (**B**) Ser235 approaches closest of all residues in the ensemble, coming to within 5 Å of NSP in one confirmation as shown. (**C**) A potential π-cation interaction between the Phe235 mutation present in the Ncap Alpha variant was modelled.

Several of the mutations in Ncap map to this linker region so we examined how they might affect these interactions with NSP3. This latter structure includes residues Ser235, Arg203, Gly204, Gly201 and Ser235 but there would appear to few direct NSP3 interactions (Figure 3A) as they mostly are located at a considerable distance from the NSP3 protein as shown in the NMR ensemble (Figure 3A). Ser235 is a possible exception to this (Figure 3B). In one conformation of the complex, this residue approaches NSP3 to within 5 Å of Lys38. In Alpha Ncap, this residue is mutated to a phenylalanine. We modelled this change into the complex which was then energy minimised revealing a possible π-cation interaction between the phenylalanine and the ε-amino group of the lysine residue. Depending on the environment, π-cation interactions can be stronger than salt bridges, providing some potential stabilisation of the complex [45] in this mutant.

Mutations in Ncap could alter its ability to bind nucleic acid. However, predicting the impact of amino acid substitutions on such interactions is made difficult as only limited information on the structure of Ncap bound to RNA is available. A structure of residues within the isolated N-terminal domain, (residues 48–171 of pdb code 7XWZ) bound to RNA does not indicate any direct interactions between the altered amino acids discussed above and the nucleic acid bound as they are not present in the structure determined by CryoEM [2]. As both N and C-terminal domains are implicated in RNA binding, which involves dimerisation/oligomerisation, it is possible that mutations in Ncap could alter interactions with their substrates [46]. However, a study examining RNA-binding in different corona virus nucleocapsids concluded that three predominant SARS-CoV-2 variants: Gamma, Delta, and Omicron as well as nucleocapsid from other corona viruses showed no overt changes in the RNA binding [47]. Similarly, no major impact of Ncap mutation on RNA binding was observed in a separate study which examined representative mutations from a large number of SARS-CoV-2 variants [48].

We carried out preliminary molecular dynamics simulations to gain insight into potential impact the amino acid substitutions might have on Ncap flexibility (Supplementary Figures S2 and S3). Analysis of an ensemble of structures obtained by molecular dynamics simulations [49] for each of the variant Ncaps revealed no large changes to the structural stability of the proteins. However, some differences between the original Ncap and variants were detected. The sum of the atomic root-mean-square fluctuation across the MD trajectory for Ncap and each variant were 701 Å for Ncap, 725 Å for the B.1.1, 650 Å for Alpha, 685 Å for Delta and 621 Å for Omicron. These variations are shown in Supplementary Figures S2 and S3 along with differences between predicted MD trajectories for each residue in the variants compared with the original sequence.

### Protein production

Synthetic genes encoding Ncap and its variants were codon-optimized by commercial suppliers (see Supplementary Table S1) using their proprietary algorithms. Creating codon-optimized genes is a well-trodden path to improve protein production levels [50,51]. There are many online tools which essentially reverse-translate the target amino acid sequence into DNA. They account for codon usage, or bias, in the host organism, as well as maintaining an appropriate GC content, avoiding repetitive sequences and undesirable secondary structures in the transcribed mRNA [52]. At the simplest level, this includes avoiding rare codons which tend to slow down translation rates. For example, arginine has six possible codons but they are not all used with the same frequency in all organisms. In *E. coli* these six codons range from the rarely used (AGG [4%] AGA/CGA [both 7%], CGG [11%]) to the most frequent (CGC/CGT [36%]) [53].

The individually cloned genes were expressed using the pT5P expression vector developed in our laboratory which was derived from plasmid pTTQ18 [54]. It has a strong but inducible bacteriophage T5 promoter [55] under the control of the *lacI^q^* repressor which is also encoded on the plasmid. The plasmid also contains a consensus Shine-Delgarno sequence (AGGAGG) [56] to ensure efficient translation initiation. To reduce the likelihood of aggregation of the expressed proteins, we reduced the temperature of the culture to 20°C upon induction as this is thought to aid in expression of soluble proteins [57].

**Figure 4.**
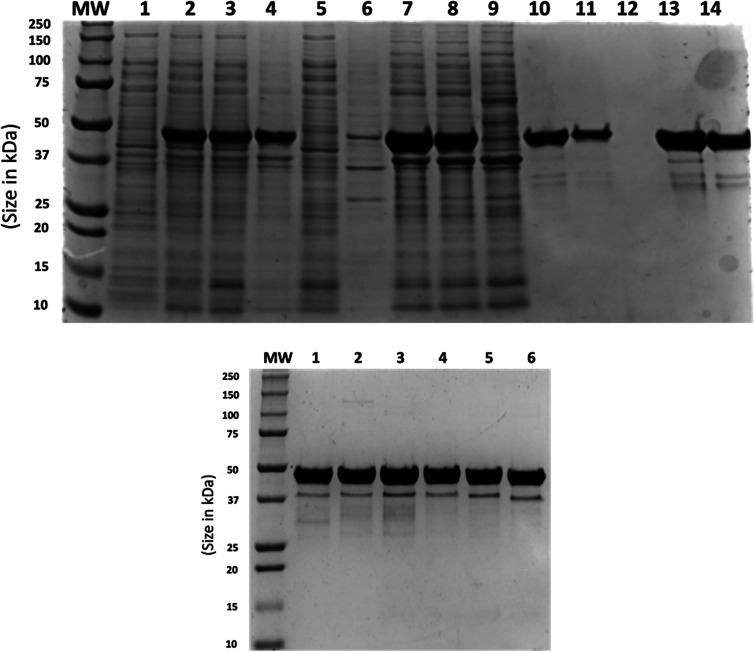
Expression and purification of 6His-tagged SARS-CoV-2 Ncap Omicron. SDS–PAGE (10%) analysis of samples from example purification. Molecular weight markers (MW, Bio-Rad (Cat. 161-0362)) are shown for each panel. Left panel shows total SDS-cell lysates of uninduced and induced *E. coli* BL21 carrying plasmid pT5P_MHTOmicronNcap (Lanes 1 and 2 respectively). The soluble fraction from induced cells after lysis, sonication and centrifugation (Lane 3). Pellet from 5% to 17% ammonium sulfate precipitation (Lane 4) and corresponding supernatant (Lane 5). The ammonium sulfate-protein pellet was resuspended in HisTrap loading buffer, centrifuged to separate into insoluble material (Lane 6) and the soluble fraction (Lane 7) and loaded on a HisTrap column (Lane 8). Lane 9 shows proteins passing through the HisTrap column. Pooled fractions eluted from the HisTrap column (Lane 10) were diluted with cation exchange loading buffer and loaded on SP Sepharose column (Lane 11). Flow through from SP column (Lane 12). Peak fractions from linear gradient elution (Lanes 13 and 14). Right panel shows samples of purified 6His-tagged; Ncap Omicron (Lane 1); Ncap Delta (Lane 2); Ncap Alpha (Lane 3); Ncap 203/204 (Lane 4); Ncap (Lane 5) as well as untagged Ncap (Lane 6).

As the T5 promoters are recognised by *E. coli* RNAP polymerase, this system can be used in most commonly available host strains. We used BL21 [58] for the work described here in combination with media based on that described by Studier for the expression of the Ncap proteins [59]. All Ncaps were expressed in soluble form and the purification procedure outlined above produced proteins which were >95% pure as exemplified for the Omicron variant (Figure 4, others shown in Supplementary Figure S4). This contrasted to previous reports in which recombinant SARS-CoV-2 Ncap was obtained as insoluble aggregates in *E. coli* requiring purification under denaturing conditions followed by refolding [13,38,60] or required fusion with maltose-binding protein to aid production of soluble material [61] followed by proteolysis of the fusion partner. Ncap and variants were obtained at yields of 7–10 mg or more of purified protein per g of cell mass from fermentations yielding 20–30 g of cells per litre of culture grown in modified Studier medium [59].

A differential ammonium sulfate precipitation (5–17% w/v) removed significant quantities of *E. coli* host cell proteins before further liquid chromatography (LC) steps. Further purification was carried out by immobilised metal ion affinity chromatography (IMAC) followed, in the case of the 6His-tagged proteins and cation exchange chromatography by elution with imidazole or salt gradients, respectively.

All proteins were shown to be free of significant nucleic acid contamination as shown by UV spectroscopy (Supplementary Figure S5). We noticed that Ncap proteins had a tendency to co-purify with nucleic acids as monitored by the samples’ *A*_260 nm_/*A*_280 nm_ ratios. It was particularly important to wash the 6His-NCAP proteins bound to the IMAC column with sufficient high-salt buffer to remove the co-purifying nucleic acids, requiring up to 20 column volumes depending on the sample, or in the case of the native protein, using PEI precipitation for the same purpose. Mass spectrometry (electrospray) showed that each protein displayed the expected mass within experimental error (Supplementary Table S3).

### Identity of minor contaminants in recombinant Ncap

As we required our Ncap proteins for immunological assays, we were keen to understand the level of purity obtained and gain insight into the nature of the inevitable contaminating *E. coli* proteins which would be present, albeit at low levels. Thus, a more detailed analysis of our 6His-tagged and untagged Ncap proteins was carried out to identify the contaminating bands that became apparent when purified samples were over-loaded on SDS–PAGE gels. Supplementary Figure S6 shows SDS–PAGE gels with overloaded purified samples. Minor bands with both higher and lower molecular weights than the main expected product were observed. However, when these bands were excised from the overloaded gels and subjected to proteomic analysis via tryptic digest with quantification estimated using intensity based absolute quantification (iBAQ) [62] all the contaminant bands appeared to have the recombinant nucleocapsid as their main constituent (>90% for all minor bands analysed). In both the untagged and tagged Ncap, the main band on the gel was comprised of over 97% recombinant Ncap as estimated by iBAQ. Human keratins, proteomic workflow contaminants, were also observed but were excluded from the analysis. The observation that the minor high-MW contaminants observed on SDS–PAGE at high loading levels also appeared to be composed mostly of Ncap could be explained by incomplete denaturation due to overloading. Alternatively, they could represent a very small fraction of the material that had undergone liquid-liquid phase separation and was resistant to SDS-denaturation.

The contaminants identified were all from *E. coli* BL21 with the most frequent/abundant being Elongation factor Tu (EF-Tu, UNIPROT accession number A0A140NCI6). This is not surprising as EF-Tu is the most highly expressed gene product in *E. coli* [63]. The next highest-level contaminant was an uncharacterised protein (A0A140SS81), followed by a Type VI secretion system effector (A0A140N758), and an alcohol dehydrogenase GroES domain protein (A0A140N870), transcriptional regulator LacI (A0A140NB96) which is encoded on the expression plasmid used in our work, methylmalonyl-CoA mutase (A0A140N835), sulfate ABC transporter (A0A140N7X7) and another transcriptional regulator, IclR (A0A140NF03). All contaminants over 0.1% are shown in Supplementary Figure S6.

### Response of Ncap variants with pooled antisera

**Figure 5.**
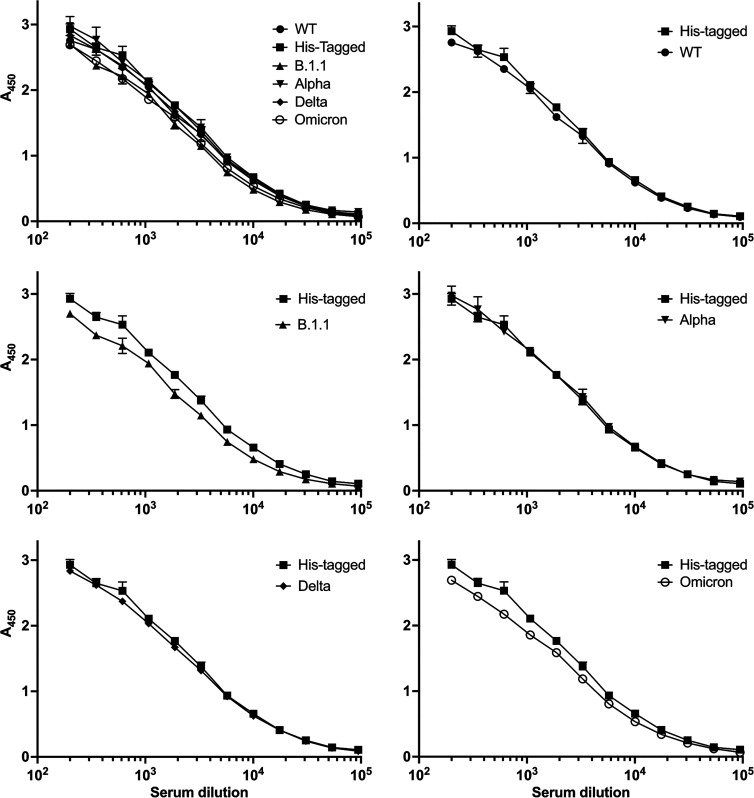
ELISA assay results for nucleocapsid variants with pooled SARS-CoV-2 positive human sera. Ncap variants were immobilised on micro-titre plates and ELISA performed as described above (*n* = 2). Top left panel compares all Ncaps produced. Serum was diluted from 1/200 by eleven 1.75-fold serial dilutions. The original wild-type SARS-CoV-2 Ncap (untagged) and the his-tagged version are compared (top right). The remaining panels show pairwise comparisons between the latter and other 6His-tagged variants.

We have previously validated an assay for the presence of human anti-SARS-CoV-2 Ncap antibodies using serum from SARS-CoV-2-confirmed cases and pre-pandemic serum samples [64]. We next compared the detectability of Ncap variants using pooled human sera (Figure 5).

There was no significant difference between the measured ELISA signal response curves for the tagged and untagged original SARS-CoV-2 recombinant proteins, nor for the Delta or Alpha [65]. However, the Omicron and B.1.1 variants elicited lower ELISA signals from the pooled serum (*P* < 0.001 in both cases). For B.1.1 the median reduction was 18.5% (range 7.8–35.5%) while for Omicron, the median reduction was 14.0% (range 7.6–36.0%). The largest reduction in signal occurred at the lowest serum concentrations tested. Further details of the analyses are presented in Supplementary Table S4.

**Figure 6.**
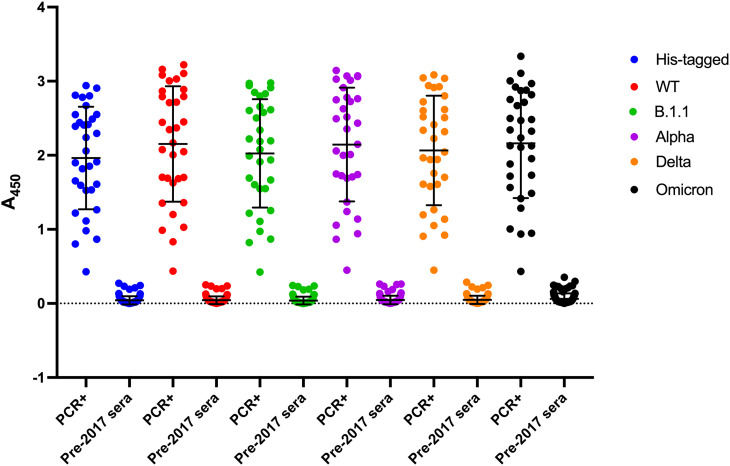
Nucleocapsid proteins elicit weak response from pre-pandemic sera. Ncap variants were immobilised on micro-titre plates and ELISA performed as described in Materials and Methods. All 32 serum samples testing positive for SARS-CoV-2 by PCR (PCR+) exhibited higher signal levels than the 94 pre-pandemic samples in these assays. Means and SD are indicated.

We also tested all our proteins for cross-reactivity with a panel of 94 pre-pandemic control sera and compared them with 32 PCR-confirmed SARS-CoV-2 positive convalescent serum samples. Figure 6 shows very low ELISA signals were obtained from the pre-pandemic samples compared with the SARS-CoV-2 positive controls. The pre-pandemic ELISA signal means for the six recombinant Ncaps tested were in the range 0.039–0.062 AU (SD range 0.052–0.074). In contrast, the corresponding signal means from the individual convalescent serum samples were 1.964–2.160 AU (SD range 0.692–0.779) for the six immobilised Ncaps.

## Conclusion

We have developed generic protocols for production of soluble, immunologically competent Ncap proteins from *E. coli* without the need for denaturing conditions or refolding. The expression vector developed is portable as the synthetic T5 promoter is recognised by native sigma 70 of *E. coli* RNA polymerase [66]. Thus, in principle it can be used in any *E. coli* strain unlike e.g. T7 expression systems which require the presence of the T7 RNA polymerase gene within the host genome or supplied on an additional genetic element [67]. Yields of up to 10 mg purified protein per gramme of cell paste were readily achieved, though we acknowledge that elements of the expression and purification protocols could be optimised for individual proteins to improve yields even further. All the molecules produced were recognised by pooled sera from confirmed SARS-CoV-2 convalescent patients although the B.1.1 and Omicron variants showed slightly reduced ELISA responses.

## Materials and methods

### Bioinformatics and structure manipulation

Phyre2 [68] was used to model structures of Ncap based on a 5 Å CryoEM model 8FD5 [2]. The PyMOL Molecular Graphics System, version 2.5.2 Schrödinger, LLC was used to render 3D visualisations, to analyse mutations and for alignment of Phyre-2 generated structures and YASARA (YASARA Biosciences GmbH) was used to carry out energy minimisation of structures. The CABSFLEX server's default parameters were used to compare protein flexibility in the variant sequences with the original by molecular dynamics simulations [49].

### Expression constructs

Synthetic genes or gene fragments were designed for optimal expression in *E. coli* (Supplementary Table S1). Genes were codon-optimised for the original SARS-CoV-2 Ncap (accession number YP_009724397) and its variants (B.1.1, QIQ08827; Alpha, QYU76755; Delta, UAL04655 and Omicron, UFO69287) in *E. coli* using standard molecular biological methods [69]. Briefly, two synthetic gene fragments were purchased from Genewiz GmbH (Germany) and designated Ncap_*N-*terminal and Ncap*_C-*terminal. They contained a 25 bp overlap at their 3′ and 5′ ends respectively. They were mixed at an equimolar ratio and assembled into a complete coding sequence using SOE PCR amplification method [70] with either forward primer Ncap_for or Native_Ncap_for and reverse primer Ncap_rev (see Supplementary Table S2) to generate the N-terminal six-histidine tagged Ncap fragment or native construct lacking the tag. The resulting PCR products contain unique EcoRI and NdeI sites upstream of the coding regions and a downstream HindIII site as shown (Supplementary Table S1). The PCR fragments were digested with restriction endonucleases HindIII and either NdeI or EcoRI, for native or 6His-tagged Ncap fragment, respectively and then cloned into expression vector pT5P digested with the corresponding enzymes.

This generated two clones designated pT5P_Ncap and pT5P_MHTNcap encoding the untagged and 6His-tagged proteins, respectively. Additionally, a variant encoding both Arg203Lys and Gly204Arg (B.1.1) with the N-terminal 6His-tag was produced by PCR using standard procedures. Briefly, the pT5P_MHTNcap plasmid was amplified with primers Ncap_for and Ncap_203/204_rev or Ncap_203/204_for and Ncap_rev (Supplementary Table S2), to generate two fragments. These fragments were gel purified and mixed in equimolar ratio and PCR amplified with Ncap_for and Ncap_rev to generate 6His-tagged Ncap 203/204 fragment for restriction endonuclease-mediated cloning with *Eco*RI and *Hind*III into pT5P using standard methods generating the plasmid pT5P_MHTNcap_B11 which encoded B.1.1 Ncap sequence (Lys 203 and Arg 204). The Alpha and Delta, variants of Ncap, encoding the same mini-His tag at their N-termini were custom synthesised (NBS Biologicals Ltd, U.K.) with codon optimisation flanked by EcoRI/NdeI and HindIII sites and supplied cloned in pUC57. The synthetic genes were inserted into expression vector pT5P using restriction enzymes EcoRI and HindIII generating plasmids pT5P_MHTAlphaNcap and pT5P_MHTDeltaNcap, respectively by standard methods [69]. The Omicron sequence was derived by inserting a synthetic gene fragment carrying the Omicron variation (custom synthesised by GeneWiz GmbH) between the NdeI and XbaI sites of pT5P_MHTNcap_B11, generating pT5P_MHTOmicronNcap. All insert sequences were confirmed by DNA sequencing by the University of Sheffield's Core Genomics Facility or GeneWiz GmbH. Sequence of all the synthetic genes and fragments supplied and the proteins they encode are presented in Supplementary Table S1.

### Protein expression

Production of recombinant protein from these plasmids was performed using standard methods. *E. coli* BL21 competent cells were transformed with the appropriate plasmid and grown on MDG agar plates [59] containing 100 µg/ml carbenicillin as follows. A freshly transformed single colony was used to inoculate 5 ml MDG media [59], supplemented with 1% vegetable-derived tryptone (Sigma 16922) and 100 μg/ml carbenicillin at 37°C, with shaking for 6 h. The starter culture was used to inoculate fresh media containing 4% vegetable-derived tryptone, 2.5% yeast extract, 25 mM Na_2_HPO_4_, 25 mM KH_2_PO_4_, 50 mM NH_4_Cl, 5 mM Na_2_SO_4_, 2 mM MgSO_4_, 0.5%, glycerol (v/v), 0.05% glucose (w/v), and 200 μl of trace-metal solution per litre (Teknova, T1001). Typically, 500 ml cultures were grown in 2.5 l baffled flasks with a drop of antifoam (A6426, Sigma) at 37°C with vigorous shaking until they reached an absorbance of *A*_600_ ∼3–4. Protein expression was induced by addition of isopropyl β-d-1-thiogalactopyranoside (IPTG, supplied by Melford U.K.) at a final concentration of 0.5 mM and the temperature lowered to 20°C for 24 h to allow accumulation of the expressed protein. Alternatively, cells were grown in a small fermenter as follows: The fermenter was prepared using 10% vegetable tryptone and 5% yeast extract autoclaved in 2 l water. Separately autoclaved solutions of 100 ml 50xM (1.25 M Na_2_HPO_4_, 1.25 M KH_2_PO_4_, 2.5 M NH_4_Cl and Na_2_SO_4_), 10 ml 1 M MgSO_4_, 1 ml 1 M CaCl_2_ were added together with sterile filtered 100 g 50% glycerol 50 ml 10% glucose, 5 ml carbenicillin (100 mg/ml) and 1 ml of trace elements solution (Teknova, T1001). The fermenter was topped up to 4.6 l with sterile water, warmed up, and 1 ml of antifoam (A6426, Sigma) was added just before the inoculation. A freshly transformed single colony was used to inoculate 5 ml MDG with carbenicillin (Studier) medium and grown at 37°C to a density of *A*_600_ 0.9–1.0. It was then transferred to 400 ml MDG with carbenicillin and grown in a shake flask to a cell density of *A*_600_ 0.9–1.0 and used to inoculate the fermenter, which was stirred at ∼500 rpm and aerated with 5 l air per minute. The entire 400 ml inoculum was used to bring the volume to 5 l and cells were grown at 28°C, doubling roughly every 60 min. Induction was carried out at a cell density ∼*A*_600_ 3 by adding 0.5 ml of 1 M filter sterilised IPTG (final concentration 0.1 mM), and 10 ml of 10% lactose was added. Cells were harvested at a cell density equivalent to ∼*A*_600 _= 25, which corresponded to ∼150 g of cell paste from a 5 l fermentation broth.

### Protein purification

LC was carried out on either an ÄKTA Prime or ÄKTA PURE system (Cytiva). Cell pellets (typically 10 g per batch) were resuspended in 50 ml in lysis buffer (25 mM Tris–HCl pH 8, 100 mM NaCl, 5% v/v glycerol, 1 mM 4-(2-aminoethyl) benzenesulfonyl fluoride hydrochloride) and lysed by the addition of 10 mg hen egg white lysozyme (Sigma, L6876) and sodium deoxycholate (Acros Organics) to a final concentration of 0.5 mg/ml then incubated overnight at 4°C. Subsequent procedures were carried out at room temperature (RT). After sonication to reduce viscosity using short bursts (∼20–30 s) in an MSE Soniprep 150 Plus sonicator, the suspension was centrifuged at 30 000 × ***g*** for 30 min and the supernatant was recovered for immobilised metal affinity chromatography.

#### 6His-tagged Ncap proteins

The supernatant was adjusted to 5% (w/v) ammonium sulfate, mixed gently and centrifuged as above. The pellet was discarded. The supernatant was adjusted to 500 mM with solid NaCl and 17% w/v with ammonium sulfate to selectively precipitate the Ncap at RT. The pellet was recovered by centrifugation as above and resuspended in 30 ml loading buffer (20 mM HEPES pH 7.8, 200 mM NaCl, 20 mM imidazole, 5% v/v glycerol) for purification by IMAC. Protein was loaded onto 2 × 5 ml HisTrap HP (Cytiva) columns connected in tandem and washed with wash buffer (20 mM HEPES pH 7.8, 2 M NaCl, 20 mM imidazole, 5% v/v glycerol) until the *A*_260 nm_/*A*_280 nm_ ratio indicated bound nucleic acids were removed. Fractions (2 ml) were collected over a 10-column volume imidazole gradient (20–500 mM, in 20 mM HEPES pH 7.8, 500 mM NaCl, 5% v/v glycerol). The purest fractions were diluted 10-fold into cation exchange loading buffer (20 mM HEPES pH 8, 10 mM NaCl, 1 mM EDTA, 5% v/v glycerol) for all protein except Delta, in which case the buffers were adjusted to 1 mM in DTT to keep the cysteine residues reduced before chromatography on a 20 ml HiPrep SP FF 16/10 column (Cytiva).

Protein was eluted over a linear gradient (10–1000 mM NaCl) and 2 ml fractions were collected. Samples were analysed by SDS PAGE on 10% polyacrylamide gels. The purest fractions were collected and buffer exchanged with storage buffer (20 mM HEPES pH 8, 120 mM NaCl, 1 mM EDTA, 10% v/v glycerol [augmented with 1 mM DTT for the Delta variant]) by ultrafiltration using Amicon Ultra-15 10 kDa cut-off centrifugal filters and flash frozen in liquid nitrogen for long-term storage at −80°C.

#### Untagged Ncap protein

Cells were lysed as above except that the buffer also contained 1 mM EDTA. After centrifugation the cell lysate was subjected to sonication to reduce viscosity and adjusted to 5% (w/v) in ammonium sulfate. Sufficient 5% polyethyleneimine-HCl (PEI, pH 8) was added to precipitate nucleic acids and mixed gently on a roller for 30 min prior to centrifugation at 30 000 × ***g*** for 30 min to remove the precipitated RNA/DNA/PEI pellet. Ncap protein was selectively precipitated by gradual addition of solid ammonium sulfate with gentle stirring to a final concentration of 17% (w/v) in presence of 500 mM NaCl. The pellet, consisting largely of precipitated Ncap was recovered by centrifugation as above and resuspended in 200 ml of cation-exchange loading buffer (20 mM HEPES pH 8, 10 mM NaCl, 1 mM EDTA, 5% v/v glycerol) and loaded on to a 20 ml HiPrep SP FF 16/10 (Cytiva) column. Protein was eluted over a 20-column volume linear gradient (10–1000 mM NaCl) and 2 ml fractions were collected. Samples were analysed by SDS PAGE on 10% polyacrylamide gels. The purest fractions were concentrated and loaded on to a HiLoad Superdex 200 16/600 120 ml (Cytiva) column for size exclusion chromatography and eluted in 25 mM Tris pH 8, 120 mM NaCl, 1 mM EDTA, 5% glycerol (v/v). The purest fractions were collected and stored at −80°C by flash freezing in liquid nitrogen.

### Mass spectrometry analysis

Analytical services were provided by the Faculty of Science biOMICS Facility, and Dept of Chemistry, University of Sheffield, and the Michael Barber Centre for Collaborative Mass Spectrometry, University of Manchester U.K. All protein samples were buffer exchanged into 50 or 200 mM ammonium acetate (Fisher Scientific 10365260) as indicated using Zeba Micro Spin columns (#89877, 75 µl) prior to mass spectrometry analysis. Intact mass analyses were performed on a Waters Vion IMS Qtof connected to an Acquity I-Class LC system. Protein samples were separated using an Acquity UPLC BEH C4 column (p/n 186004496), maintained at 80°C. The system was controlled using UNIFI software in positive ion mode with a capillary voltage of 2.75 kV and source temperature of 150°C. The LC gradient was developed at a flow rate of 0.2 ml/min over 10 min as follows: 0 min: 5% B, 1 min, 50% B 3.5 min: 95% B 7.5 min, 5% B, 10min (mobile phases A: water (MQ)/0.1% formic acid and mobile phase B (100% acetonitrile/0.1% formic acid). Data analysis was performed using UNIFI. Native mass spectrometry was carried out using direct-infusion nano-electrospray ionisation from in-house pulled borosilicate capillary tips on a Waters Synapt G2-S in positive ion mode, capillary voltage of 1.1–1.4 kV, desolvation temperature of 80°C and cone voltage of 10 V. Data analysis was performed using MassLynx and Origami software.

#### Serology

IgG-specific responses of serum samples to Ncap proteins were assessed by ELISA assays exactly as reported previously [64]. IgG response curves were generated using pooled serum collected from two hospitalised patients following PCR-confirmed SARS-CoV-2 infections. Individual patient responses were generated using serum collected prior to 2017, or from healthcare workers following PCR-confirmed SARS-CoV-2 infections. Ncap proteins were immobilised in microtitre plates (Immulon 4HBX; Thermo Scientific, 6405) at 4°C overnight at 2 μg/ml (50 µl/well) in PBS (pH 7.4). Plates were washed with 0.05% PBS-Tween, then blocked for 1 h at RT with 200 μl/well 0.5% casein buffer. The IgG response curves were generated in duplicate by serially diluting in 1·75× steps from an initial 1:200 dilution of pooled sera. Individual patient samples were tested in duplicate wells at a single dilution of 1:200. Samples were loaded at 100 µl/well and incubated for 2 h at RT, followed by washing and addition of 100 µl/well of goat anti-human IgG-HRP conjugate (Invitrogen, 62-8420) at 1:500 dilution, and incubation for 1 h at RT. Wells were then washed and 100 µl/well TMB substrate (KPL, 5120-0074) was added and left to develop for 10 min. Stop solution (KPL, 5150-0021) was added at 100 µl/well, and the absorbance read at 450 nm. Anonymised serum samples from hospitalised COVID-19 patients, and those collected prior to 2017 during routine clinical care were obtained with approval from the Sheffield Teaching Hospitals’ Research and Development office (Sheffield, U.K.). Serum samples from healthcare workers following SARS-CoV-2 infections were collected as part of the COVID-19 Humoral ImmunE RespOnses in front-line HCWs (COVID-19 HERO) study. Following internal scientific review, local R&D (5 May 2020 ref: STH21394) and HRA and Health and Care Research Wales approval were given (29 April 2020 ref: 20/HRA/2180, IRAS ID: 283461).

## Data Availability

Supporting Tables and Figures are available in the Supplementary Materials. Raw mass spec data from which contaminating proteins were identified are available at doi:10.15131/shef.data.25041743.

## Open Access

Open access for this article was enabled by the participation of University of Sheffield in an all-inclusive *Read & Publish* agreement with Portland Press and the Biochemical Society under a transformative agreement with JISC.

## Abbreviations

iBAQ: intensity based absolute quantification
IMAC: immobilised metal ion affinity chromatography
LC: liquid chromatography
Ncap: nucleocapsid protein
NSP3: non-structural protein 3
RAT: rapid antigen test
RT: room temperature
SARS-CoV-2: severe acute respiratory syndrome coronavirus 2
